# Academic Helplessness and Life Satisfaction in Korean Adolescents: The Moderated Mediation Effects of Leisure Time Physical Activity

**DOI:** 10.3390/healthcare11030298

**Published:** 2023-01-18

**Authors:** Mihye Kim, Kyulee Shin, Sanghyun Park

**Affiliations:** 1 Institute of Sports Science, Seoul National University of Science & Technology, Seoul 01811, Republic of Korea; 2 Department of Sports Sciences, Seoul National University of Science & Technology, Seoul 01811, Republic of Korea; 3 Department of Sport for All, Korea National Open University, Seoul 03087, Republic of Korea

**Keywords:** leisure time physical activity, academic helplessness, learned helplessness, depression, life satisfaction, adolescents

## Abstract

This study examined whether depression mediates a relationship between academic helplessness and life satisfaction and whether the mediating effect differs depending on participation in leisure time physical activity (LTPA) from a sample of 2384 middle school students in South Korea. Identifying these factors could help in developing intervention strategies for promoting life satisfaction. Structural equation modeling analyses were employed to understand how various factors influence adolescents’ life satisfaction. First, the effect of academic helplessness on life satisfaction was mediated by depression. Second, the mediating effect of depression was moderated according to participation in LTPA: the size of the negative mediating effect of depression on the relationship between academic helplessness and life satisfaction was reduced in the LTPA group compared to the non-LTPA group. The current findings suggest that encouraging engagement in physical activity may be a crucial vehicle for affecting academic helplessness, depression, and life satisfaction among early adolescents. Implications and future directions are discussed.

## 1. Introduction

The overall life satisfaction of adolescents continues to decline [1]. East Asian adolescents, in particular, exhibited lower levels of life satisfaction than their North American and European counterparts [2]. Previous studies suggested that academic pressure contributed to adolescents’ low life satisfaction in East Asia [3,4]. Despite the economic expansion, East Asian nations such as Korea, China, and Hong Kong continue to retain competitive education systems [5]. These nations have a social atmosphere emphasizing academic work, and graduating from prestigious universities is a prerequisite for good jobs, high wages, and high social status [6,7]. Consequently, not only parents but also students themselves have high academic expectations and study-related stress [8,9].

Academic stress among East Asian adolescents has reached a level that requires professional treatment and support and has morphed into “learned helplessness” [10,11]. It falls well beyond the simple dimension of tension or anxiety. Learned helplessness is a concept proposed in 1967 by Martin Seligman and Obermeyer in animal experiments. The dogs of the experimental group learned that they could not stop receiving an electric shock no matter what they tried. As a result, they did not try to escape when they were shocked, but only whined. These subjects were considered to have acquired learned helplessness [12]. Learned helplessness is defined as a psychological phenomenon in which one continuously faces a painful and uncontrollable situation and despairs instead of avoiding or overcoming it, even though one can avoid or overcome it with one’s own ability [13].

Over the past decade, research on the learned helplessness of Korean teenagers has been widely documented [14,15]. In the past, most high school students were prone to extreme academic stress. They attended private academies and institutes after their regular school hours and on weekends to maintain their studies and rank among classmates [16,17]. Unfortunately, this “education fever” has now spread to middle school students. As expected, these young adolescents have a very difficult time dealing with this increased pressure and lack of leisure time at such a tender age [18,19,20]. Meanwhile, when Koreans enter middle school, the number of their physical education classes decreases. The seventh and eighth graders have three per week, and the ninth graders have two. This is due to an increase in mandatory academic and elective classes [21]. Despite the decrease in physical activity opportunities for middle school students, unfortunately, Korean parents do not enroll these children in outside physical extra-curricular activities such as community sports. At this age, their college admission is more valued [22].

Unsatisfied test results and test anxiety may lead to learned helplessness in a school setting, according to research findings. Adolescents’ test anxiety was positively associated with learned helplessness in school [23]. Early adolescents’ low subjective satisfaction with academic achievement directly affected academic helplessness [24]. Elkadri [25] also found that parents’ and teachers’ pressure regarding academic achievement was associated with adolescents’ learned helplessness.

Accordingly, Korean scholar Bak and his colleagues developed an academic helplessness scale based on Martin Seligman’s theory of learned helplessness [26]. While Seligman’s learned helplessness is a question about the overall characteristics of human psychology, academic helplessness is limited to academic situations (e.g., exams, classes, etc.). Therefore, in order to specifically demonstrate the characteristics of helplessness in academic situations, separate verification is necessary because the measurement range is different for learned helplessness as opposed to academic helplessness. Specifically, it is needed to identify the relationships between academic helplessness, depression, and life satisfaction. Depression is a common symptom of learned helplessness [27] and is a strong representative factor that lowers adolescents’ life satisfaction [28]. Therefore, it is reasonable to assume that depression may play a mediating role between learned helpless and life satisfaction. Previous research has examined associations between learned helplessness and life satisfaction [29], between learned helplessness and depression [27], and between depression and life satisfaction [28], respectively; however, these three factors have not been examined jointly in previous research.

To treat learned helplessness, it is vital to eliminate the habit of negative thinking and to enhance the learned helplessness defense mechanisms. For instance, cognitive-behavioral therapy [30] and emotional support from parents or instructors are beneficial [31]. Also, it has been said that reaching small, attainable goals is a good way to boost protective factors like self-efficacy [32]. Specifically, physical exercise is widely recognized as an essential way to strengthen protective qualities such as self-efficacy [33]. In addition, recent research indicated that during the COVID-19 pandemic, those who participated in leisure-time physical exercises had considerably lower levels of learned helplessness than those who did not [34]. These findings imply that physical exercise may prevent or moderate the development of learned helplessness.

Therefore, based on previous studies, we hypothesized: (1) depression mediates the relationship between academic helplessness and life satisfaction; (2) participation in LTPA moderates the mediation effect of depression between academic helplessness and life satisfaction.

### 1.1. Depression as a Mediator in the Academic Helplessness–Life Satisfaction Relationship

Academic helplessness is a concept of learned helplessness in academic situations based on the learned helplessness theory presented by Seligman in 1976 [35]. Academic helplessness occurs after students repeatedly experience stressful situations. When students regularly perform poorly on exams even after studying hard, they start to believe that they are incapable of success, so they give up trying [26].

Unlike elementary school students, East Asian middle school students experience higher academic stress due to the region’s scholastic ranking system; absolute grading is no longer used [36,37]. In addition to an increase in the amount of studying and academic evaluation changes, high parental academic pressure has also been found to be a strong influencer on academic helplessness among Asian middle school students [37,38].

A typical symptom of learned helplessness is depression [27]. As a result of academic helplessness, students may experience a feeling of out of control and depression [39]. In other words, learned helplessness is closely related to low self-esteem [40], and low self-esteem predicts depression [41]. Therefore, learned helplessness should be identified early.

Depression increased due to academic helplessness may also affect adolescents’ life satisfaction [42]. Depression is well-known to negatively affect adolescents’ life satisfaction [28]. Previous studies have reported that academic helplessness and life satisfaction have a negative correlation [43,44]. While previous studies have examined associations between academic helplessness, depression, and life satisfaction separately, collaborative relationships between academic helplessness, depression, and life satisfaction have not been researched. Therefore, we hypothesized that depression would mediate relationships between academic helplessness and life satisfaction.

### 1.2. LTPA as a Moderator in the Academic Helplessness, Depression and Life Satisfaction Relationship

Previous studies suggested that LTPA participation can affect learned helplessness (reference). Buffart et al. [45] concluded that exercise participation increases the subject’s mastery of the activity and alleviates learned helplessness through self-efficacy. Mastery is having control over the forces that affect one’s life, and improving mastery through exercise can help improve cognitive distortions caused by repeated failure experiences [45,46].

Physical activities in adolescents help prevent and reduce the incidence of depressive symptoms [47]. Hrafnkelsdottir et al. (2018) reported that more frequent vigorous physical activities in adolescents lowed the risk of depressive symptoms, low self-esteem, and life dissatisfaction [48]. McMahon et al. (2017) also showed that more frequent physical activities and participation in leisure sports led to a lower level of depressive symptoms and greater well-being in adolescents [49].

## 2. Materials and Methods

### 2.1. Participants

The purpose of this study was two-fold. First, the structural relationship between adolescents’ academic helplessness, depression, and life satisfaction was analyzed using the Korea Children and Youth Panel Survey 2018 (KCYPS 2018). Second, the differential effects of LTPA participation in this structural relationship were also analyzed.

To obtain a representative sample, KCYPS 2018 applied a stratified sampling method. Face-to-face surveys using tablet PCs were conducted. The data consisted of seventh-grade middle schoolers (13-year-olds). In total, 2384 data points were analyzed. Initially, there were 2590 surveys, but 206 were incomplete and were thus excluded. The demographic characteristics of participants classified by gender are shown in Table 1, since there is no meaningful difference in the mean of the three variables (academic helplessness, depression, and life satisfaction) by gender.

### 2.2. Measures

#### 2.2.1. Academic Helplessness

Academic Helplessness Scale by Bak et al. [26] was used. This scale was developed based on Seligman’s Learned Helplessness theory and related studies [12,50]. This particular scale, unlike general learned helplessness scales that are used to measure adults in general situations [50], focuses on students’ learned helplessness in academic situations [26]. Academic helplessness occurs after a student repeatedly experiences a stressful situation. When students regularly perform poorly on exams even after studying hard, they start to believe that they are incapable of success, so they give up trying. This scale consists of 16 items under four sub-factors including lack of locus of control, lack of academic motivation, lack of positive affect, and lack of active engagement. Lack of locus of control means that it is difficult to believe that one’s attitude and effort can achieve positive results. Lack of academic motivation means that the motivation to control the process and results related to academic achievement in a positive direction is low. Lack of positive affect means that as a result of academic helplessness, students experience emotions such as loss of pleasure and depression. Lack of active engagement means that dependent and passive behaviors appear in academic situations [26]. The scale is a 4-point Likert scale ranging from 1 = not true at all to 4 = very true. Higher scores indicated a higher likelihood of academic helplessness. Sample questions from the academic helplessness scale include, “No matter how hard I try, my grades will not improve”, ”I have no motivation to study”, “I am losing interested in studies”, “I don’t concentrate on studying even during an exam period”.

#### 2.2.2. Depression

Kim, Kim, and Won’s (1984) Depression Scale was used [51]. This scale has ten items in the Symptom Checklist-90-Revised (SCL-90-R), developed by Derogatis (1977) [52]. Sample questions from the academic helplessness scale include, “I have a lot of worries”, “I often blame myself when things go wrong”, “I feel lonely”. The scale is a 4-point Likert type scale ranging from 1 = not true at all to 4 = very true. Higher scores indicated a higher likelihood of depression.

#### 2.2.3. Leisure Time Physical Activity

Leisure Time Physical Activity (LTPA) was measured using a questionnaire asking about one’s duration of exercise that produced sweat during the previous week. Students answered on a 5-point Likert scale for this item (0 = not at all to 4 = 4 h or more). This questionnaire has been proven valid by its frequent use in the literature, such as the National Medical Checkup Questionnaire [53], the International Physical Activity Questionnaire [54], and the Student Physical Activity Measurement Sheet [55].

#### 2.2.4. Life Satisfaction

Life Satisfaction was measured with a Korean version of the Satisfaction with Life Satisfaction (SWLS) developed by Diener et al. [56]. It contained five items, including, “In general, my life is close to my ideal”, “The circumstances of my life are very good”, and “I am satisfied with my life”. Measurements were made on a 4-point Likert scale (1 = strongly disagree to 4 = strongly agree).

### 2.3. Analysis

A confirmation factor analysis (CFA) was performed to verify the relationship between the observed variables and the basic structure in the measurement model. To be specific, a two-step approach by Anderson and Gerbing (1988) was applied [57]. We first evaluated a measurement model through CFA and then evaluated a research model through a structural equation model (SEM). In this process, academic helplessness was combined into four parceled variables based on the user guide of KCYPS 2018. To evaluate measurement and structural models, we used multiple indexes, such as chi-square, the Steiger-Lind Root Mean Square Error of Approximation (RMSEA), the Tucker-Lewis index (TLI), and the comparative fit index (CFI). Composite reliability (CR) and average variance extracted (AVE) were calculated for the components of each measurement scale to verify convergent validity, discriminant validity, and reliability. Lastly, structural equation modeling was carried out to test the proposed two hypotheses.

## 3. Results

### 3.1. Descriptive Statistics

Our measurement model was evaluated by CFA using AMOS 24.0. The results revealed a suitable fit to the data (χ² = 1455.362, df = 149, *p* = 0.000, TLI = 0.925, CFI = 0.941, RMSEA = 0.058), as shown in Table 2.

Basically, convergent validity was evaluated by the criteria recommended by Fornell and Larcker [58]. The CRs and AVEs for each latent variable should exceed the thresholds of 0.7 and 0.5, respectively. The result of our study showed that the CRs ranged from 0.839 to 0.828, and AVEs ranged from 0.494 to 0.569. It means that the AVE of life satisfaction had a lower value than the criteria. However, the value of 0.4 is acceptable because if the AVE value is less than 0.5 but the composite reliability is higher than 0.6, the convergent validity of the construct is acceptable [58]. In the case of discriminant validity, it is acceptable if the square root of AVE for each latent variable exceeds all correlation coefficients among the latent variables. As shown in Table 3, the minimum value of the square root of AVEs was 0.702. Discriminant validity was also achieved because this value was higher than the maximum value of the correlation coefficient (*r* = 0.545).

### 3.2. Testing the Moderated Mediating Models

Based on a measurement model which proved validity and reliability, SEM was conducted to test the hypothetical causal relationships among the three latent variables. The result revealed that the structural model was acceptable (χ² = 1455.362, *df* = 149, *p* = 0.000, TLI = 0.925, CFI = 941, RMSEA = 0.058). The results of the research hypothesis are shown in Table 4. According to Table 4, three paths among academic helplessness, depression, and life satisfaction were significant. Specifically, academic helplessness had a positive influence on depression (*b* = 0.834, *p* < 0.001), depression had a negative influence on life satisfaction (*b* = −0.322, *p* < 0.001), and academic helplessness influenced life satisfaction negatively (*b* = −0.176, *p* < 0.001).

In order to statistically test Hypothesis 1, the mediation effect of depression between academic helplessness and life satisfaction was performed using the bootstrapping method. As a result, depression had a significant mediation effect between academic helplessness and life satisfaction (*b* = −0.269, *p* < 0.001).

Also, as a result of analyzing whether these mediation effects differed depending on participation in LTPA, the mediation effect of depression in the non-LTPA group (*b* = −0.360, *p* < 0.001) was greater than the mediation effect of depression in the LTPA group (*b* = −0.227, *p* < 0.001). Then, to statistically test Hypothesis 2, we used the ‘MyModMed’ package of AMOS to test whether the mediation effect of each model had a significant difference (moderated mediation effect). As a result, a significant difference was found. The mediation effect of the LTPA group was less than that of the non-LTPA group. It means that LTPA participation of adolescents contributed to lowering the negative effect of academic helplessness.

## 4. Discussion

This study sought to identify whether depression mediates the relationship between academic helplessness and life satisfaction of middle school students, and whether this mediating effect is moderated by participation in LTPA.

First, it was shown that academic helplessness not only negatively impacted life satisfaction directly but also negatively impacted life satisfaction via depression. In other words, it may be said that middle school students’ academic helplessness had a detrimental impact on their depression levels, which lowered their degree of life satisfaction. According to these results, academic helplessness may have a significant role in determining the level of life satisfaction experienced by students through either direct or indirect pathways.

The findings of this research have implications for clinical practice. It is important that the goal of reducing depressive symptoms be included in school-based intervention programs designed for middle school students with academic helplessness. Previous research on learned helplessness has shown that it is strongly associated with reactive depression [59,60].

Unlike endogenous depression, reactive depression is a temporary symptom of depression due to a relatively specific stress event [61,62]. Therefore, middle school students can improve depressive symptoms by improving their sense of accomplishment or self-efficacy. Encouraging participation in their favorite extracurricular and school activities or participation in physical activities with parents, family, and peers might assist [63].

Second, it was found that the mediating effect of depression on the relationship between middle school students’ academic helplessness and life satisfaction differed according to the presence or absence of participation in LTPA. The mediating effect of depression on the link between academic helplessness and life satisfaction was less in the group that did physical activities in their free time than in the group that did not. These findings are consistent with previous studies [64,65] that middle school students’ participation in physical activities is a key factor in depression. In addition, Bélair et al. (2018) reported that inactive adolescents were more likely to be depressed and anxious as opposed to physically active adolescents [66]. The results of this study also concluded that, like previous studies, middle school students participating in physical activities were less stressed [67] and had higher life satisfaction [68].

Based on prior research, it is reasonable to speculate how LTPA influences academic helplessness. Participation in LTPA is a crucial means of experiencing positive emotions among adolescents [69]. According to Fredrickson’s broaden and build theory of positive emotions, the experience of positive emotions contributes not only to feeling good but also to reducing negative thinking (e.g., I won’t be able to do well) by promoting positive automatic thinking [70]. Positive emotions, according to Fredrickson, help reduce depression and increase life satisfaction by expanding the momentary thought-action repertoire [71].

In addition, LTPA improves self-efficacy which is a protective factor for learned helplessness and depression [72]. Prior literature reported that people who experienced learned helplessness could overcome this by trying to achieve small goals frequently in lieu of feeling overwhelmed by larger or occasional ones [73]. Additionally, the support of significant others in their every life of their accomplishment is vital. Therefore, we present that small goals and tasks offered throughout physical activity can help build mastery and self-control, reduce academic helplessness, and increase East Asian adolescents’ life satisfaction [45,74]. For example, instead of a student’s initial tennis lessons’ goals being to collect points successfully, the student must first learn how much force to apply to one’s swing to hit the ball over the net without going out of bounds. Once this is mastered, the student can progress to more advanced skills and strokes, eventually winning a match.

## 5. Conclusions

One of the primary implications of this study is that LTPA can be a major resolution to alleviate adolescents’ depression due to high academic helplessness and can increase life satisfaction. Therefore, it is necessary to increase LTPA participation among adolescents who experience academic helplessness. However, considering Trout and Graber’s (2009) research that showed difficult tasks in physical activities and physical education class situations, mastery-oriented tasks should be provided as an opportunity to achieve small goals [75].

Second, teenagers in East Asian countries often start private education to prepare for college entrance exams in earnest after graduating from elementary school [5]. Therefore, due to this extreme pressure, parents cannot simply be suggested to enroll their children in physical activities. In this regard, policy alternatives such as expanding physical education classes in schools and restricting operating hours of private education on weekends should be enacted.

There are a few limitations of this study. First, demographic and sociological factors (such as parenting and teachers’ support) were not controlled. In future studies, such variables need to be considered. Second, the level of LTPA engagement was measured by a single scale (the duration of participating in LTPA). The scale needs to be specified into sub-types, such as the types of physical activity participation (competitive activities or non-competitive cooperative activities) in future studies. Despite such limitations, this study is meaningful in that it suggested specific treatments for East Asian adolescents, considering this population’s relatively low level of life satisfaction. In addition, using national-level data, the empirical basis for participation in physical activities was presented as a means of preventing and treating academic helplessness, depression, and low life satisfaction.

In conclusion, depression mediates the relationship between academic helplessness and life satisfaction among adolescents. Clinical assessment and treatment should include goals to reduce depressive symptoms in middle school students with academic helplessness. Furthermore, participation in LTPA moderates the mediation effect of depression between academic helplessness and life satisfaction. Encouraging engagement in physical activities may be a crucial vehicle for adolescents with academic helplessness.

## Figures and Tables

**Table 1:** Characteristics of participants.

Variables		Male (*n* = 1278)		Female (*n* = 1106)	
		*n*	%	*n*	%
Age (years)	12	6	0.5	3	0.3
	13	1259	98.5	1086	98.2
	14	13	1.0	17	1.5
Subjective health	very unhealthy	10	0.8	10	0.8
	unhealthy	100	7.8	91	8.2
	healthy	667	52.2	636	57.5
	very healthy	501	39.2	369	33.5
Leisure Time Physical Activity (LTPA)	none	320	25.0	572	51.7
	1 h	341	26.7	280	25.3
	2 h	246	19.2	136	12.3
	3 h	120	9.4	63	5.7
	4 h and more	251	19.6	55	5.0
**Variables**		**Mean (SD)**		**Mean (SD)**	
Academic helplessness		1.966 (0.507)		1.954 (0.519)	
Depression		1.733 (0.580)		1.857 (0.624)	
Life satisfaction		2.652 (0.556)		2.598 (0.538)	

**Table 2:** Confirmatory factor analysis for measurement model.

	λ	AVE	CR
Academic helplessness		0.569	0.839
Lack of locus of control.	0.607		
Lack of academic motivation.	0.799		
Lack of positive affect.	0.818		
Lack of active engagement.	0.775		
Depression		0.526	0.917
I feel down.	0.689		
I feel unfortunate or sad, and depressed.	0.791		
I have a lot of worries.	0.630		
I feel like I want to die.	0.690		
I cry often.	0.661		
I often blame myself when things go wrong.	0.709		
I feel lonely.	0.762		
I lack interest in everything.	0.747		
I don’t think I have a bright future.	0.751		
I find everything difficult.	0.801		
Life satisfaction		0.494	0.828
In general, my life is close to my ideal.	0.670		
The circumstances of my life are very good.	0.780		
I am satisfied with my life.	0.788		
I’ve accomplished something important to me.	0.665		
If I could live my life again, I wouldn’t change a thing.	0.590		

Chi-square = 1455.362, df = 149, *p* = 0.000, TLI = 0.925, CFI = 0.941, RMSEA = 0.058.

**Table 3:** Correlation matrix for all measurement variables.

	Academic Helplessness	Depression	Life Satisfaction
Academic helplessness	(0.754)		
Depression	0.545 ***	(0.725)	
Life satisfaction	−0.351 ***	−0.465 ***	(0.702)

Note. Square root of AVEs is in parentheses. *** *p* < 0.001

**Table 4:** Hypotheses testing using bootstrapping method.

Path	b	SE	*t*	*p*
Variable relationships				
Academic helplessness **→** depression	0.834	0.043	19.426	<0.001
Depression **→** life satisfaction	−0.322	0.024	−13.35	<0.001
Academic helplessness **→** life satisfaction	−0.176	0.035	−5.018	<0.001
Hypotheses 1. Mediation effects of depression				
Academic helplessness **→** depression **→** life satisfaction	−0.269	0.024	−11.033	<0.001
Hypotheses 2. Moderated mediation effects				
Mediation effect of depression in non-LTPA group (a)	−0.360	0.050	−7.125	<0.001
Mediation effect of depression in LTPA group (b)	−0.227	0.020	−7.985	<0.001
Moderated mediation effects (a–b)	−0.133	0.064	2.078	0.037

## Data Availability

Data is available upon request at https://www.nypi.re.kr/archive/mps (accessed on 22 October 2022).
